# Case report: A novel *de novo* variant of *NACC1* caused epileptic encephalopathy and intellectual disability

**DOI:** 10.3389/fpsyt.2024.1446698

**Published:** 2024-10-03

**Authors:** Jiahao Wu, Jing Gan, Yimin Hua, Yifei Li, Di Qie

**Affiliations:** Key Laboratory of Birth Defects and Related Diseases of Women and Children of Ministry of Education (MOE), Department of Pediatrics, West China Second University Hospital, Sichuan University, Chengdu, Sichuan, China

**Keywords:** NACC1, WES, intellectual disability, epileptic encephalopathy, neurological developmental disorder

## Abstract

**Background:**

Genetic disorders could also contribute to intellectual disability. Using whole exome sequencing (WES), several variants have been identified as autosomal-dominant inheritance intellectual disability. Thus, the application of WES has demonstrated its critical role in distinguishing intellectual disability in children patients, which provides essential diagnosis and promotes therapeutic strategy.

**Case presentation:**

The proband, an 18-month-old female patient, presented with a complex clinical profile characterized by profound developmental delay, epilepsy, and neurological developmental impairment. WES identified a heterozygous c.913A>G variant in exon 2 of *NACC1*, resulting in disease caused by a change in the amino acid sequence, affecting the protein features and resulting in splice site changes, as revealed by MutationTaster analysis. The protein structure of NAC1 was built and named AF-Q96RE7-F1, and the mutant site was beyond the BTB/POZ, NLS, and BEN domains. Subsequently, PyMOL software was used to illustrate the molecular structure between the wild type and the mutant type of NAC1. The residues around the 304 site of amino acid changed in NAC1 p.T304A with an altered hydrogen bond, indicating an unstable structure. The patient was diagnosed with intellectual disability and profound developmental delay with epilepsy harboring a novel *de novo NACC1* variant. Upon hospital admission, a comprehensive treatment regimen was initiated, including antiseizure medications, nutritional supplements, and rehabilitation training. As a result, the patient’s movement performance improved. However, recurrent epilepsy attacks still occurred.

**Conclusion:**

This is the first case revealing a novel *NACC1* c.903A>G variant that induced a neurological impairment in an infant. This report expanded the understanding of the non-domain-associated variant of *NACC1* and developmental disorder.

## 1 Introduction

The presentation of intellectual disability in children is a critical concern in clinical management. The prevalence of intellectual disability varies widely, ranging from 8.7 to 36.8 per 1,000 live births (1). Due to the heterogeneity of intellectual disability, its frequency ratio differs globally. The diagnosis of intellectual disability typically involves identifying clinical phenotype symptoms such as delayed or slowed learning of any kind, difficulties with reasoning and logic, and problems with judgment and critical thinking (2, 3). Notably, the majority of documented cases present with mild intellectual disability characterized by deficits in adaptive behavior, often associated with socioeconomic status, the prognosis of which is closely related to the education level and the treatment received by affected children. Better socioeconomic status would have provided advanced education and training intervention for patients with intellectual disability, which could lead to improved clinical outcomes. However, severe and moderate intellectual disability, indicated by significant reductions in various scales of evaluation, such as an IQ < 50, should generally be treated as a pathogenic condition. On the other hand, mild intellectual disability would be identified among the population as several environmental factors are involved in its assessment. Moreover, a part of mild intellectual disability should still be treated as pathogenic. Identification of the etiology of such impairments is essential to improve prognosis. Environmental factors, such as brain damage or neuronal injuries during the perinatal period, including intracranial infection, tumors, neonatal asphyxia, and hypoxic–ischemic encephalopathy, are significant contributors to intellectual disability. In addition, genetic disorders play a crucial role in the etiology of intellectual disability (4).

With the rapid development of next-generation sequencing (NGS), personalized genome sequencing and the identification of potential variants can now be completed in a short duration with high accuracy (5). In the last decade, an increasing number of studies have provided novel insights into the genetic disorders associated with intellectual disability. In most developed countries, autosomal-dominant (*de novo* variants) disorders predominantly affect pediatric neurological development. However, in some underdeveloped regions, autosomal-recessive inherited intellectual disabilities are more prevalent due to the high incidence of inbreeding (2, 6). An approach combining NGS and bioinformatics is used to identify novel intellectual disability genes and to screen candidate genes. NGS has efficiently expedited the research on intellectual disability and has provided new strategies at the clinical level in recent years. The earliest genetic tests used in the investigation of intellectual disability included karyotyping, which identifies aneuploidies, such as Down syndrome (trisomy 21) and Edwards syndrome (trisomy 18), as well as large structural rearrangements such as insertions, deletions, and duplications (7). In addition, copy number variations (CNVs) causing *de novo* and inherited mutations have been associated with intellectual disability, such as the disorder (OMIM #612001) involving a microdeletion on 15q13.3 (8–10). Using whole exome sequencing (WES), *ARID1B*, *SYNGAP1*, *DYRK1A*, *MED13L*, *KCNQ2*, *CTNNB1*, *STXB1*, *KMT2A*, *PACS1*, *FOXP1*, and *SMARCA2* have been found to be the most commonly mutated autosomal-dominant intellectual disability genes (11, 12). Some of these genes are crucial for neuronal differentiation in the developing brain and play important roles in synaptic formation and transmission. Currently, around 180 genes or loci, which are involved in autosomal-dominant intellectual disability, have been reported in the literature. Thus, the application of WES has demonstrated its critical role in distinguishing intellectual disability in pediatric patients, providing essential diagnostic information and promoting therapeutic strategies.

Herein, we report on an 18-month-old female patient carrying a novel *de novo* heterozygous c.913A>G variant in exon 2 of *NACC1* who presented with a complex clinical profile characterized by severe intellectual disability, profound developmental delay, epilepsy, and neurological developmental impairment.

## 2 Methods

This study was approved by the Ethics Committee of the West China Second Hospital of Sichuan University (approval no. 2021-069). In addition, written informed consent was obtained from the patient’s parents prior to performing WES and for the inclusion of the patient’s clinical and imaging details in publications.

The genetic test was performed at 18 months old. Peripheral blood sample was obtained from the patient using an ethylenediaminetetraacetic acid (EDTA) anticoagulant blood sample tube stored at 4°C for less than 6 h. DNA was extracted using the Blood Genome Column Medium Extraction Kit (Tiangen Biotech, Beijing, China) according to the manufacturer’s instructions. WES was performed using the NovaSeq 6000 platform (Illumina, San Diego, CA, USA), and raw data were processed using FastP to remove adapters and to filter low-quality reads. Paired-end reads were aligned to the Ensembl GRCh37/hg19 reference genome using the Burrows–Wheeler Aligner. Variant annotation was performed in accordance with database-sourced minor allele frequencies (MAFs) and practical guidelines on pathogenicity issued by the American College of Medical Genetics. The annotation of MAFs was performed based on the 1000 Genomes, dbSNP, ESP, ExAC, and Chigene in-house MAF databases and Provean, Sift, Polypen2_hdiv, and Polypen2_hvar databases using R software (R Foundation for Statistical Computing, Vienna, Austria).

## 3 Case presentation

### 3.1 Clinical presentation and physical examination

The proband in this study was an 18-month-old female patient who had a clinical constellation of profound developmental delay, severe malnutrition, epilepsy, feeding difficulty, and congenital heart disease as atrial septal defect (ASD). She suffered from recurrent pneumonia due to coughing and choking on milk since she was born.

The patient was born at 38 + 5 weeks of gestation to non-consanguineous Chinese parents and had no family history of epilepsy or disability syndromes. She had a birth weight of 2,950 g, birth length of 48 cm, occipitofrontal circumference of 33 cm, and an Apgar score of 8–10–10 at 1–5–10 min post-delivery. She was admitted to the neonatal intensive care unit (NICU) for intrauterine distress and neonatal asphyxia after birth. However, the severity of neonatal asphyxia had been evaluated as a slight level, which was considered to rarely affect her neurological development. Moreover, she presented normal adaptation in the short term in terms of feeding and family care after discharge from the neonatal department. Unfortunately, a significant psychomotor developmental delay was identified, as the patient failed to hold her head upright, to chase sounds, and to laugh in the first half year after birth. Subsequently, Bayley Scales of Infant Development revealed that both the intelligence scale and the exercise scale were impaired, equivalent only to a 2-month-old infant performance. At the age of 9 months, the patient suffered her first seizure attack, which was characterized by loss of consciousness, staring, and hypertonia of limbs. The first seizure attack occurred in a wake condition, without fever and other particular medical issues. Electroencephalography (ECG) was not performed at the first seizure. However, the initial brain MRI showed that myelination of the white matter was delayed, the corpus callosum was thin and short, and the bilateral frontal top was slightly widened. Levetiracetam (initial dosage of 10 mg/kg, twice a day) was used to treat the epilepsy, the dosage of which was increased into 30 mg/kg (twice a day) in 3 months. However, the onset of epilepsy was not controlled well. Subsequently, there were four recurrent seizure attacks at ages 11, 14, 15, and 16 months, lasting 5–40 min.

On admission, the heart rate of the patient was 110 bpm and the blood pressure measured at 86/48 mmHg. The respiratory rate was 24 breaths per minute. At the age of 18 months, her weight and length were 7,000 g (*Z*-score = −3.08) and 72 cm (*Z*-score = −3.15), respectively, and the occipitofrontal circumference was 43 cm (*Z*-score = −2.32), and she still could not talk and sit or walk alone. A severe malnutrition status was observed, with little subcutaneous fat. The ophthalmologic examination revealed no abnormality. There was no surface wound, indicating the absence of accidental injuries. Physical examination of the chest and abdomen did not reveal any abnormalities. The heat beats were regular and strong. The patient did not present any neurological pathological signs, including Babinski, Chaddock, and Oppenheim signs. The extremities were warm, with a capillary refill time of 3 s. The muscle strength and tension in the upper extremities were normal, while the muscle strength was lower in bilateral lower extremities. Furthermore, no signs of pathological or meningeal irritation were observed.

In addition, the patient’s parents denied any positive family history of tumors, intellectual disabilities, cardiac attacks, hypertension, or coronary artery diseases. There was also no reported history of diabetes or obesity among family members. No inherited diseases, including neurological diseases, cardiomyopathies, or metabolic disorders, were identified in the family history.

### 3.2 Laboratory and imaging evaluation

Routine blood examination and hepatic and renal function demonstrated normal ranges. Blood tests revealed elevated ammonia (60.5 μmol/L; normal value, <20 μmol/L), β-hydroxybutyrate (1.59 mmol/L; normal value, <0.27 mmol/L), and carboxylate anion (182.1 μmol/L; normal value, 20–100 μmol/L). Thyroid function tests indicated low thyroid-stimulating hormone (TSH) and free thyroxine (FT4) levels. The nucleic acid and autoantibody tests ruled out Epstein–Barr virus, hepatic viral infections, and Coxsackie virus. The cerebrospinal fluid (CSF) examination results were normal.

**Figure 1:**
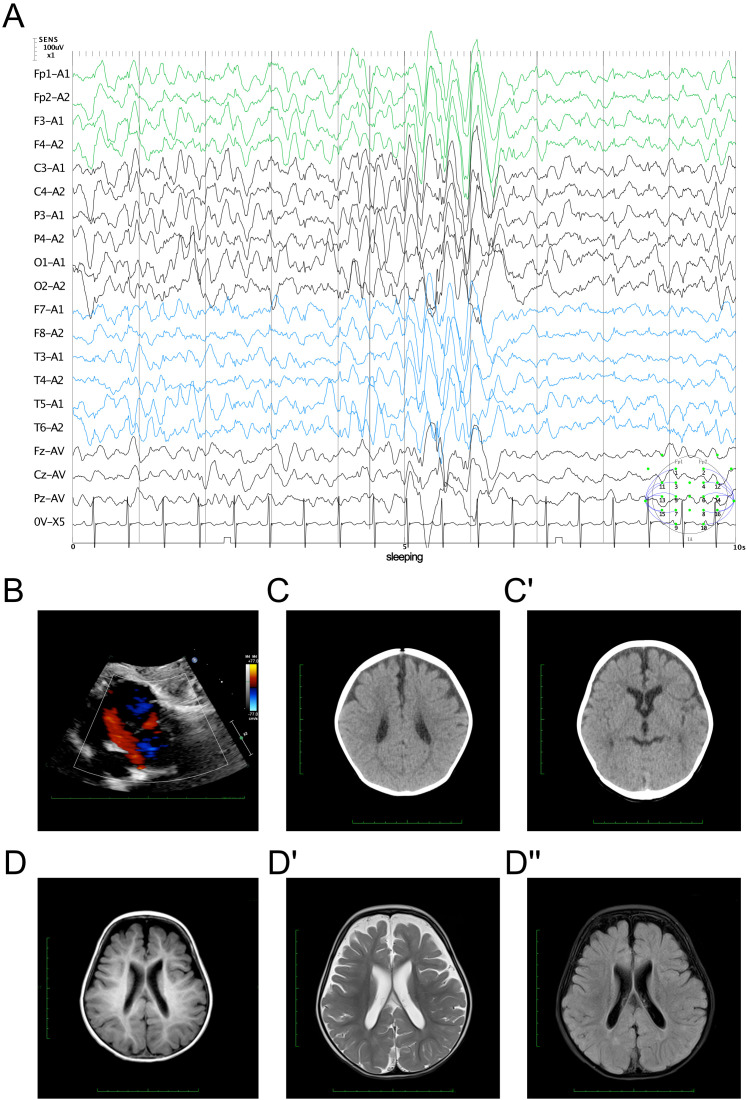
Clinical manifestation of the proband. **(A)** Electroencephalography findings showing bilateral slow wave activity mixed with spike waves during sleep stages I–II. **(B)** Echocardiography results demonstrating an atrial septal defect (ASD). **(C, C′)** Brain computed tomography results showing a widening bilateral frontotemporal extracerebral space. **(D, D")** Cerebral magnetic resonance imaging evaluation. The cerebral hemisphere surface demonstrated irregularities **(D)** with reduced volume in the bilateral frontal parietal lobes and with widened extracerebral and ventricular spaces on the T2 signal **(D′)** with dark fluid **(D")**.

The EEG findings indicated sleep stages I–II with bilateral, fully-guided slow waves mixed with spike waves and occasional spike discharges (**Figure 1A**). The cardiac ultrasound results suggested an ASD (superior and central), mild pulmonary stenosis, and normal left ventricular systolic function (**Figure 1B**). The brain computed tomography results presented a widening bilateral frontotemporal extracerebral space (**Figure 1C**). Brain MRI revealed reduced volume in the bilateral frontal and parietal lobes, with widened extracerebral spaces of the bilateral and third ventricles (**Figure 1D**). The cerebral hemispheres exhibited more tortuous surfaces and thickened blood vessels.

### 3.3 Molecular results

**Figure 2:**
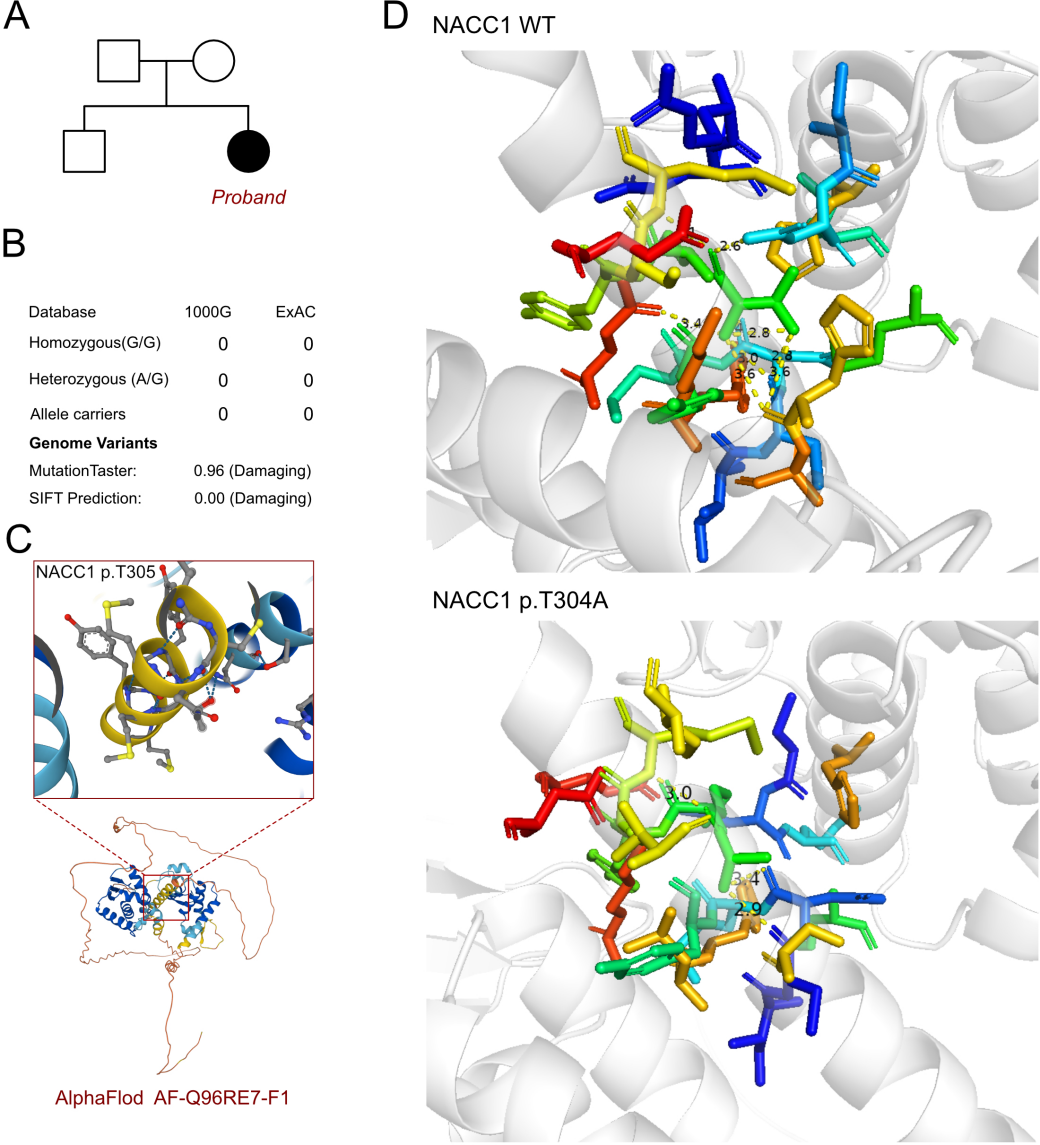
Molecular features of *NACC1*. **(A)** The proband exhibited a *de novo* heterozygous variant of *NACC1* (c.913A>G, p.T305A). **(B)** The c.913A>G variant of *NACC1* has never been reported in 1000G and ExAC and predicted protein damage through MutationTaster. **(C)** The protein structure of NAC1 was built and named AF-Q96RE7-F1. **(D)** Relationship of the residues around the mutant site indicating the change in structure due to the variant of *NACC1* (p.T305A).

As an inherent neurological disorder was suspected, a genetic test was performed to explore any associated variants. WES was performed for this proband and her parents. The analysis results of WES identified a *de novo* heterozygous variant, i.e., c.913A>G (p.T305A), of the *NACC1* gene (**Figure 2A**). Such variant was not inherited from the maternal or the paternal side. Furthermore, the c.913A>G variant of *NACC1* has never been reported in a database, hence was considered as a novel variant (**Figure 2B**). In addition, we excluded all the potential variants involved in neurological and muscular disorders. Subsequently, we reviewed all the other variants that have been reported as pathogenic or likely pathogenic, but none of them was confirmed to be associated with the phenotype of the proband, i.e., severe intellectual disability and profound developmental delay, severe malnutrition, epilepsy, and feeding difficulty. Therefore, we suspected that the novel heterozygous variant of *NACC1*, c.913A>G, contributed to the pathogenic phenotype of this proband as a complicated clinical neurological dysfunction manifestation. To elucidate the molecular architecture of the human *NACC1* gene, MutationTaster with R software was used to predict the pathogenicity of *NACC1* c.913A>G (p.T304A) and to assess the impact of this mutation on the protein structure. As there was no available full-length protein crystal structure for NAC1 (encoded by *NACC1*), which was analyzed using X-ray or cryo-EM, the AlphaFold3 protein structure software (https://alphafold.ebi.ac.uk/) tool was used to predict the protein crystal structure. The protein structure of NAC1 was built and named AF-Q96RE7-F1 (**Figure 2C**) (13, 14). Within the structure, the mutant site was beyond the BTB/POZ, NLS, and BEN domains. The region of 30–124 amino acids was involved in the BTB/POZ domain, the region of 180–207 amino acids was involved in the NLS domain, and the region of 374–471 amino acids was involved in the BEN domain. Subsequently, modeling analysis was performed using AlphaFold3 (https://golgi.sandbox.google.com/) for the mutant site in the wild type with AF-Q96RE7-F1. Thereafter, PyMOL software was used to illustrate the molecular structure between the wild type and the mutant type of NAC1. The residues around the 304 site of amino acid changed in NAC1 p.T304A, with an altered hydrogen bond, indicating an unstable structure (**Figure 2D**). According to the American College of Medical Genetics, the mutation c.913A>G has certain pathogenicity (PS2+PM2+PP2). Analyses using MutationTaster revealed that the c.913A>G variant impaired the transcription of *NACC1*, leading to the amino acid sequence and the protein features being affected, as well as to changes in the splice site, which was predicted to have caused the disease.

### 3.4 Final diagnosis and treatment

Following an analysis of the clinical manifestations, imaging assessments, and genetic screening, the patient was diagnosed with intellectual disability and profound developmental delay with epilepsy harboring a novel *de novo NACC1* variant. Upon hospital admission, a comprehensive treatment regimen was initiated, including antiseizure medications, nutritional supplements, and rehabilitation training. As a result, the patient’s movement performance improved. However, recurrent epilepsy attacks still occurred one or two times a year.

## 4 Discussion

*NACC1* encodes the nucleus accumbens-associated protein 1 (NAC1), also known as BTB/POZ domain-containing protein 14B (BTBD14B). NAC1 is a multifunctional protein that acts as a versatile transcription factor and plays a role in protein turnover. Schoch et al. were the first to associate a human Mendelian disease with a *NACC1* variant, making it a plausible disease-associated gene, as numerous neurodevelopmental disorders have been increasingly linked to misregulated gene expression (15, 16).

Initially identified and cloned as a cocaine-inducible transcript from the nucleus accumbens, NAC1 is involved in addictive behavior and reward motivation (17). In addition, it has other biological functions, including involvement in the psychomotor response to cocaine in rats and in vertebral patterning in mice; interaction with Parkin, suggesting a role in Parkinson’s disease; and involvement with TDP-43, which is implicated in amyotrophic lateral sclerosis (17, 18). The role of NAC1 in normal neurological function is underscored by its involvement in protein turnover in dendritic cells and in the maintenance of synaptic plasticity (11). As a transcriptional repressor and a protein-binding factor, NAC1 likely regulates neural development and network establishment by altering the expression, localization, and degradation of numerous downstream nervous system genes. Furthermore, NAC1 has critical roles in various human cancers, in embryonic stem cell proliferation, and in the maintenance of stemness through the direct transcriptional regulation of c-Myc (19). NAC1 is also essential for RIG-I-like receptor-mediated innate immune responses against viral infections (20, 21). Daniel et al. identified SynGAP1, GluK2A, and several SUMO E3 ligases as novel *NACC1* interaction partners in the brain (22). Komulainen-Ebrahim et al. conducted a detailed study on the levels of mitochondrial oxidative phosphorylation system (OXPHOS) complexes in cultured fibroblasts from the patient with NACC1 variant using blue native polyacrylamide gel electrophoresis (BN-PAGE) (23). The authors observed a 1.5-fold increase in the fully assembled complex I levels in patient-derived cells compared with control cells, although the in-gel activity was similar to that of the control, suggesting a decreased complex I activity in patient-derived fibroblasts. The expression of other OXPHOS complexes was normal in the patient-derived fibroblasts. Schoch et al. analyzed muscle biopsy samples from a patient with the c.892C>T *NACC1* variant, revealing a reduction in several OXPHOS complexes, including complexes I and IV.

Schoch et al. firstly reported seven individuals with profound developmental delay and/or intellectual disability, epilepsy, and feeding difficulties (24). Seizure control varied among these individuals, but generally required multiple antiseizure medications and specialized neurological care. In addition, five of the individuals presented with bilateral cataracts. Brain MRI showed delayed myelination in four individuals and decreased brain volume in six individuals. Komulainen-Ebrahim et al. described four patients with complex neurological phenotypes, including cyclic dysautonomia, profound intellectual disability, epilepsy, and feeding difficulties (23). Movement disorders were recognized between 2 months and 1 year of age. Hyperkinetic movements and spasticity or muscle hypertonia were more prominent during periods of irritability and insomnia in all patients. Several drugs, including clonidine, onabotulinum toxin A, tetrahydrocannabinol, clonazepam, and baclofen, provided mild to moderate effects. Moreover, Lyu et al. reported on a 4-year-old Chinese Han female patient with a *de novo* c.892C>T variant in *NACC1* resulting in severe intellectual disability (25). Rehabilitation training and levetiracetam therapy were provided for the patient. By age 4, she could turn over, but could not sit, walk, or talk independently. A recent study by Schoch et al. found the recurrent c.892C>T variant to be associated with a universal feature of incapacitating episodic irritability of unclear etiology among 14 affected individuals (26).

The c.892C>T variant in *NACC1* occurs within a CpG dinucleotide in an arginine codon (22, 27). This CpG pattern is linked to *de novo* events at numerous loci with advanced paternal age (23). Previous research has suggested that advanced paternal age could lead to the hotspot variant in NACC1. The c.892C>T and c.913A>G variants are located outside the BTB/POZ and BEN domains. Loss-of-function variants in the BTB/POZ domain are associated with cancer progression (28, 29), while a missense variant in the BEN domain is linked to intellectual disability. The c.892C>T variant has been identified as causing PINK1/Parkin pathway inhibition and mitochondrial injuries in neurons, leading to apoptosis and autophagy resistance, potentially triggering neuronal cell death and the onset of developmental and neurodegenerative diseases. The c.913A>G variant requires further validation. However, the novel *de novo* c.913A>G variant, which is located near the reported variant, might alter the function of the BEN domain.

## 5 Conclusions

NAC1 is a crucial protein for normal neurological function and development. Variants in *NACC1*, particularly the novel *de novo* c.913A>G variant, lead to severe neurodevelopmental disorders and intellectual disability. These findings highlight the importance of *NACC1* in neural development and its potential role in various neurodevelopmental and neurodegenerative diseases. Understanding the precise mechanisms by which *NACC1* mutations cause these conditions might offer new avenues for diagnosis and therapy.

## Data Availability

Publicly available datasets were analyzed in this study. This data can be found here: https://ngdc.cncb.ac.cn/gsa-human/browse/HRA008639.
